# Enhanced IL-1β production is mediated by a TLR2-MYD88-NLRP3 signaling axis during coinfection with influenza A virus and *Streptococcus pneumoniae*

**DOI:** 10.1371/journal.pone.0212236

**Published:** 2019-02-22

**Authors:** Angeline E. Rodriguez, Christopher Bogart, Christopher M. Gilbert, Jonathan A. McCullers, Amber M. Smith, Thirumala-Devi Kanneganti, Christopher R. Lupfer

**Affiliations:** 1 Department of Biology, Missouri State University, Springfield, Missouri, United States of America; 2 Department of Pathology, Cox Medical Center South, Springfield, Missouri, United States of America; 3 Department of Pediatrics, University of Tennessee Health Sciences Center, Memphis, Tennessee, United States of America; 4 Department of Immunology, St. Jude Children’s Research Hospital, Memphis, Tennessee, United States of America; Virginia Polytechnic Institute and State University, UNITED STATES

## Abstract

Viral-bacterial coinfections, such as with influenza A virus and *Streptococcus pneumoniae* (*S*.*p*.), are known to cause severe pneumonia. It is well known that the host response has an important role in disease. Interleukin-1β (IL-1β) is an important immune signaling cytokine responsible for inflammation and has been previously shown to contribute to disease severity in numerous infections. Other studies in mice indicate that IL-1β levels are dramatically elevated during IAV-*S*.*p*. coinfection. However, the regulation of IL-1β during coinfection is unknown. Here, we report the NLRP3 inflammasome is the major inflammasome regulating IL-1β activation during coinfection. Furthermore, elevated IL-1β mRNA expression is due to enhanced TLR2-MYD88 signaling, which increases the amount of pro-IL-1β substrate for the inflammasome to process. Finally, NLRP3 and high IL-1β levels were associated with increased bacterial load in the brain. Our results show the NLRP3 inflammasome is not protective during IAV-*S*.*p*. coinfection.

## Introduction

Secondary bacterial infections during influenza A virus (IAV) infection contribute to disease severity and mortality [1–3]. *Streptococcus pneumoniae* (*S*.*p*.) and Staphylococcus aureus (*S*.*a*.) are the dominant pathogens associated with IAV coinfection [1–3]. The coinfection of IAV and *S*.*p*. results in pneumonia due to multiple factors [4–8]. After IAV exposure, *S*.*p*. causes a severe infection requiring only a low inoculum size compared to a single infection [9]. IAV can also alter host immunological responses or lung homeostasis that can subsequently impede bacterial clearance [8, 10–13]. IAV infection enhances bacterial growth by depleting or functionally altering alveolar macrophages [11, 12, 14, 15] and dysregulating neutrophils [16–18]. These two pathogens further work in a synergistic manner to increase activation of innate immune pattern recognition receptors (PRRs), which results in enhanced cytokine production and inflammation [9, 19–21]. Because the immune response to coinfection plays an essential function in the pathology of this disease, it is important that we have a better understanding of the causes of inflammation.

Although inflammation is necessary to fight infections, dysregulated cytokine production can have a detrimental impact on pathogen clearance and also lead to immunopathology. For example, anti-inflammatory cytokines, such as IL-10, counteract excessive inflammation during IAV-*S*.*p*. coinfection [19, 21]. However, elevated IL-10 during secondary bacterial pneumonia impairs the immune response and results in increased bacterial burden [19]. Type I interferons (IFN-α/β) have important protective functions against viral replication, yet they increase bacterial burden by decreasing neutrophil responses needed to fight off bacterial pathogens [22–25]. IFN-γ production is also linked to impaired alveolar macrophage function and bacterial burden after coinfection [11, 12, 14, 26–29]. Although specific cytokines play pathological roles during coinfection, the treatment of human patients with corticosteroids during coinfection provides little benefit [30–34]. Thus, the specific roles of cytokines need to be examined in greater detail.

The inflammasome is a multiprotein complex containing an activating PRR, such as the NLR Family Pyrin Domain Containing 3 (NLRP3) protein, the adaptor protein apoptosis-associated speck-like protein containing a caspase recruitment domain (ASC) and the cysteine protease caspase-1 [35]. NLRP3 senses damage associated molecular patterns (DAMPs) like reactive oxygen species, and K^+^ and H^+^ fluxes resulting from cell damage caused by IAV or *S*.*p*. infection [36–40]. Absent in melanoma 2 (AIM2) can also activate the inflammasome when it recognizes *S*.*p*. DNA in the cytoplasm of host cells [39, 41]. Active caspase-1 in the inflammasome cleaves inactive pro-IL-1β and pro-IL-18 into their active forms and also triggers pyroptotic cell death [42–44]. Once activated, IL-1β and IL-18 leave the cell through pores and induce inflammation [42–44]. Previous research has shown that IL-1 receptor deficient mice (*Il1r1*^*-/-*^) are more susceptible to IAV-*S*.*p*. and IAV-*S*.*a*. coinfection [45, 46]. However, how IL-1β is produced and regulated during IAV-*S*.*p*. coinfection is not known. In particular, although NLRP3 and AIM2 have known roles during single infections with IAV and *S*.*p*., their importance during coinfection with these pathogens is not known [36–41].

To gain a better understanding of the regulatory pathways for the inflammasome and IL-1β during IAV-*S*.*p*. coinfection, we investigated the role for the NLRP3 and AIM2 inflammasomes both *in vitro* and *in vivo* in mice. Our data demonstrate the NLRP3 inflammasome primarily activated during IAV-*S*.*p*. coinfection. Furthermore, enhanced TLR2-MYD88 mediated priming contributes to elevated IL-1β levels. Finally, deletion of NLRP3 was associated with decreased bacterial burden in peripheral tissues and quicker weight recovery from coinfection. Whereas *Myd88*^*-/-*^ mice had lower bacterial burden in some tissues and less inflammation in the lung, but they were more susceptible to coinfection. Together, these results illuminate the contribution of the TLR2-MYD88-NLRP3 signaling axis during IAV-*S*.*p*. secondary bacterial infection.

## Materials and methods

### Ethics statement

All animal experiments were performed under MSU Animal Care and Use Committee (IACUC) protocol 16.009 in accordance with IACUC guidelines, the AVMA Guidelines on Euthanasia, NIH regulations (Guide for the Care and Use of Laboratory Animals), and the U.S. Animal Welfare Act of 1966. IACUC approval was obtained for the use of Ketamine and Xylazine for anesthesia. CO2 asphyxiation followed by cervical dislocation was the approved method for euthanasia. In addition to mice, embryonated chicken eggs (Charles River Labs) were infected with IAV at 10 days old for production of virus stocks.

### Mice

C57BL/6J, *Nlrp3*^*-/-*^, *Myd88*^*-/-*^ and *Aim2*^*-/-*^ mice were originally obtained from The Jackson Laboratory and then bred in-house at Missouri State University (MSU). All *in vivo* experiments were performed under biosafety level 2 conditions at the Missouri State University Vivarium. *Ripk2*^*-/-*^, *Trif*^*-/-*^, *Mavs*^*-/-*^, *Casp1/11*^*-/-*^, *Asc*^*-/-*^, *Tlr7*^*-/-*^, and *Tlr2*^*-/-*^ knockout mice were housed at St. Jude Children’s Research Hospital and have been reported previously [47–49].

### Infectious agents

Mouse-adapted influenza A/PR/8/34 H1N1 virus (hereafter referred as PR8) stocks were propagated by allantoic inoculation of hen’s eggs with seed virus. Plaque assays were performed using Madin-Darby canine kidney cells to confirm stock titer. Type 3 *S*.*p*. (ATCC 6303) was used in our studies. Colony Forming Units (CFU) assays were performed to confirm bacterial stock concentrations using brain heart infusion (BHI) agar.

### Cell culture

Bone Marrow Derived Macrophages (BMDMs) were generated by harvesting bone marrow from tibia and femurs from WT, or *Ripk2*^*-/-*^, *Trif*^*-/-*^, *Mavs*^*-/-*^, *Nlrp3*^*-/-*^, *Myd88*^*-/-*^, *Aim2*^*-/-*^, *Casp1/11*^*-/-*^, *Asc*^*-/-*^, *Tlr7*^*-/-*^, and *Tlr2*^*-/-*^ knockout mice, all on the C57BL/6J background. After bone marrow harvesting, cells were differentiated in L929 conditioned medium for 5 days as previously described [50]. BMDMs were then counted and seeded at a density of 1x10^6^ cells per well in 12 well plates. The following day, BMDMs were infected as described below.

### In vitro infection

BMDMs were washed 2X with phosphate buffered saline (PBS), and 200μl of RPMI was added to each well. BMDMs were then mock infected, or single infected with either 10 MOI of PR8 or 1 MOI of *S*.*p*., or coinfected with 10 MOI of PR8 then 3 hours later 1 MOI of *S*.*p*. After an additional hour, 200μl RPMI with 20% FBS was added to all wells. Cell lysates and supernatants were then collected at 6 h, 12 h or 24 h for analysis by western blot, qRT-PCR, and ELISA.

### In vivo infection

On day 0, mice were anesthetized by intraperitoneal injection with 80mg/kg Ketamine and 8mg/kg Xylazine diluted in PBS. Groups of 5–7 mice were infected with 125 PFU PR8 intranasally in a volume of 30μl PBS. Some of these groups were then mock infected and the others coinfected on day 7 with 1000 CFU *S*.*p*. intranasally in a volume of 30μl of PBS [9, 51]. Additional groups of 5–7 mice were singly infected with 1000 CFU *S*.*p*. on day 7. At each time point, mice were monitor at least daily for weight loss and mice were euthanized by CO_2_ asphyxiation followed by cervical dislocation when they achieved 30% weight loss or became moribund. Alternatively, mice were euthanized on day 9 or day 12 to collect lungs, liver, spleen and brain for examining lung pathology, cytokine levels by ELISA and flow cytometry, and for titering CFUs and PFUs. Viral titers from lungs that were homogenized by passing through a 70μm cell strainer were analyzed by plaque assay using MDCK cells as previously reported [52]. Quantification of *S*.*p*. from lung, liver, spleen and brain homogenates (also generated by passing through a 70μm cell strainer) was performed by making 10-fold serial dilutions of lung homogenate and plating 50μl on BHI agar plates and incubating in a 37°C incubator with 5% CO_2_.

### Histology

Lungs from coinfected mice collected on day 9 (2 d post-coinfection) or day 12 (5 d post- coinfection) were fixed in 10% neutral buffered formalin. Lungs were embedded in paraffin and 5μm sections stained with hematoxylin and eosin. Sections were examined and scored according to the scoring system in Table 1. Total lung pathology was the sum of all individual category scores for each animal. Histology slides were scored by Dr. Christopher Gilbert, a board certified pathologist at Cox Medical Hospital in Springfield, Missouri.

**Table 1:** Lung histology scoring metric.

Scoring	Infiltrate of neutrophils	Infiltrate of lymphocytes	Airways	Architecture
0	No significant abnormality.	No significant abnormality.	unremarkable	intact architecture
1	Minimal / focal	Minimal / focal	plugging	focal breakdown
2	Mild patchy	Mild patchy	obliteration	severe breakdown
3	Mild fairly diffuse	Mild fairly diffuse		
4	Patchy moderate	Patchy moderate		
5	Moderate	Moderate		
6	Marked infiltrate	Marked infiltrate		
Alveolar fluid	YES (+1) / NO (0)			
Necrosis	YES (+1) / NO (0)			

Table 1 provides the scoring metric used to evaluate lung histology samples. The higher the number, the more severe the pathology. Total lung pathology was the combined score for all categories for an animal.

### Enzyme-linked immunosorbent assay

Cytokine levels in cell culture supernatants or whole lung homogenates were analyzed using mouse Ready-SET-Go ELISA kits (eBioscience) for IL-1β (88–7013), IL-6 (88–7064), or TNF-α (88–7324). Assays were performed using the manufacturer’s recommendations. Microtiter plates were read at 450nm using a BioTek ELx800 microplate reader.

### Immunoblotting

Lysates collected from *in vitro* infected BMDMs at different time points as described above (*In vitro* infection scheme and collection) were subjected to SDS-PAGE and gels were electrophoretically transferred onto polyvinylidine difluoride membranes (PVDF). Protein expression was examined using the following primary antibodies: anti-β-Actin and anti-IL-1β (D6A8, D3H1Z; Cell signaling technologies) were used with anti-rabbit HRP secondary antibody (Jackson Immuno Research, 111-035-144). Membranes were incubated in SuperSignal West Femto Maximum Sensitivity Substrate (ThermoScientific, 34096) and bands were visualized using an Azure Biosystems C300 imaging system.

### Isolation of mRNA and real-time qPCR

Extraction of total mRNA was done using TRIZOL (Invitrogen). mRNA was then reverse-transcribed into cDNA using a high capacity cDNA reverse transcription kit (Applied Biosystems, 4368814). cDNA samples were analyzed by real-time quantitative PCR (RT-qPCR) using DyNAmo HS SYBR Green qPCR Kits (Thermo Scientific, F410L) and relative values normalized to β-actin control. The following primer pairs were used: β-Actin FW 5’- GGC TGT ATT CCC CTC CAT CG-3’, Rev 5’-CCA GTT GTT AAC AAT GCC ATG T-3’. IL-1β FW 5’ GAC CTT CCA GGA TGA GGA CA -3’, Rev 5’ AGC TCA TAT GGG TCC GAC AG-3’, TNF-α FW 5’-CAT CTT CTC AAA ATT CGA GTG ACA A- 3’, Rev 5’-TGG GAG TAG ACA AGG TAC AAC CC-3’, IL-6 FW 5’- TCC AGT TGC CTT CTT GGG AC -3’, Rev 5’- GTA CTC CAG AAG ACC AGA GG -3’.

### Flow cytometry

Lungs from coinfected mice were collected on day 9 (2 d post-coinfection) and passed through a 70μM cell strainer. Cells were isolated by centrifugation through a 35% percoll solution and then stained with a 1:200 dilution of each antibody to determine which cell types express pro-IL-1β in the lung during coinfection. An initial surface stain was performed using anti-CD11b, anti-CD11c, anti-Gr1, anti-CD3ε, and anti-CD19 (TONBO biosciences, clones M1/70, N418, RB6-8C5, 145-2C11, 1D3). Then, cells were fixed with IC Fixation Buffer and permeabilized in 1x Permeabilization Buffer (eBioscience, 00-8222-49, 00-8333-56) followed by staining with anti-IL-1β pro-form (eBioscience, clone NJTEN3). Cells were distinguished based on the following gating strategies: CD3ε—CD19^-^ CD11b^+^ CD11c^-^ Gr1^-^ (macrophages), CD3ε—CD19^-^ CD11b^+^ CD11c^+^ Gr1^-^ (Dendritic cells), CD3ε^-^CD19^-^CD11b^+^CD11c^-^Gr1^+^ (neutrophils and inflammatory monocytes), CD3ε^+^CD19^-^ (T cells), CD3ε ^-^CD19^+^ (B cells), and lineage negative cells were considered mainly epithelial cells.

### Statistical analysis

Student’s t-test, one-way and two-way ANOVA with Tukey’s or Dunn’s post hoc analysis were performed using PRISM 6 from Graphpad. For weight loss during *in vivo* experiments, two-way ANOVA with Dunnett’s post hoc analysis was performed using PRISM 6. *In vivo* survival analysis was performed using the Wilcoxon test using PRISM6. A p value <0.05 was considered statistically significant. Data are graphed as the mean +/- the SEM.

## Results

### Cell types producing IL-1β during IAV and *S*.*p*. coinfection

**Fig 1:**
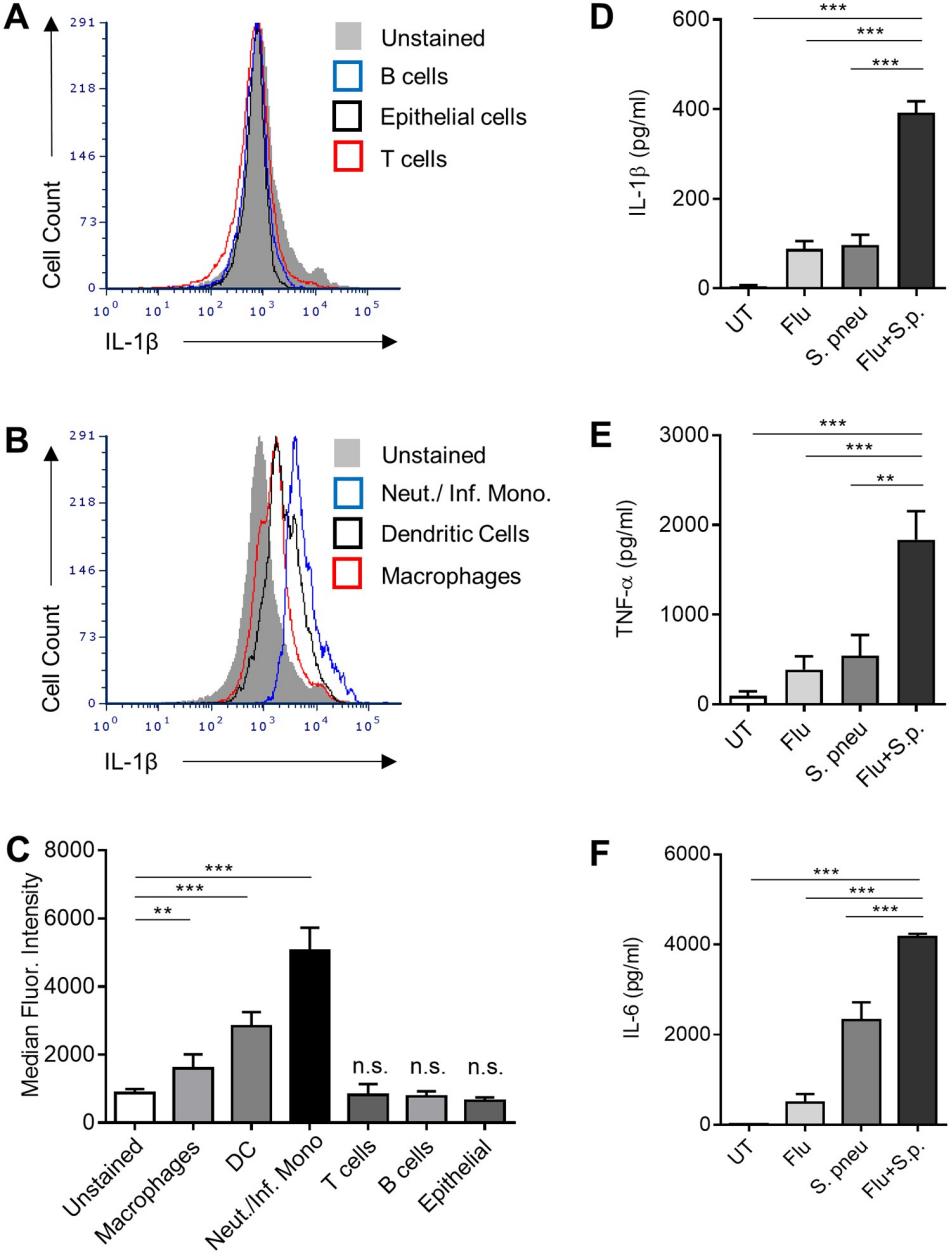
Increased production of cytokines *in vitro* during coinfection. **(A-C)** IL-1β protein expression in cells from lungs of coinfected mice was determined by flow cytometry. **(D-F)** Samples collected from WT BMDMS 24 hours post-infection were examined by ELISA for IL-1β, TNF-α and IL-6. Data are pooled from 2–5 independent experiments with n = 2–3 wells per experiment. One-way ANOVA using Tukey’s post hoc analysis was used for statistical comparison (Mean +/- SEM). ns: not significant, p values: <0.01 (**), <0.001 (***).

To determine the mechanisms by which coinfection of IAV and *S*.*p*. affect the inflammasome and IL-1β, we first examined cells from the lungs of coinfected mice to determine which cell types produced IL-1β. We found that only CD3ε^-^ CD19^-^ CD11b^+^ CD11c^-^ Gr1^-^ (macrophages), CD3ε^-^ CD19^-^ CD11b^+^ CD11c^+^ Gr1^-^ (Dendritic cells), and CD3ε^-^ CD19^-^CD11b^+^ CD11c^-^ Gr1^+^ (neutrophils and inflammatory monocytes) produced pro-IL-1β on day 2 post-coinfection (Fig 1A–1C). CD3ε^+^ CD19^-^ (T cells), CD3ε^-^ CD19^+^ (B cells), and lineage negative cells (mainly epithelial cells) showed no expression of pro-IL-1β (Fig 1A–1C). Based on these results, we elected to use bone marrow derived macrophages (BMDMs) to examine the signaling pathways responsible for IL-1β production *in vitro*. BMDMs were infected with influenza A/PR/8/34 H1N1 (PR8) and *S*. *pneumoniae* ATCC 6303 type 3 strain (*S*.*p*.) either alone or 3 h apart. After 24 h, significant increases in the levels of IL-1β, IL-6 and TNF-α were observed from BMDMs coinfection with IAV and *S*.*p*. compared to untreated or single infected samples (Fig 1D–1F).

### The NLRP3 inflammasome controls IL-1β activation during coinfection

**Fig 2:**
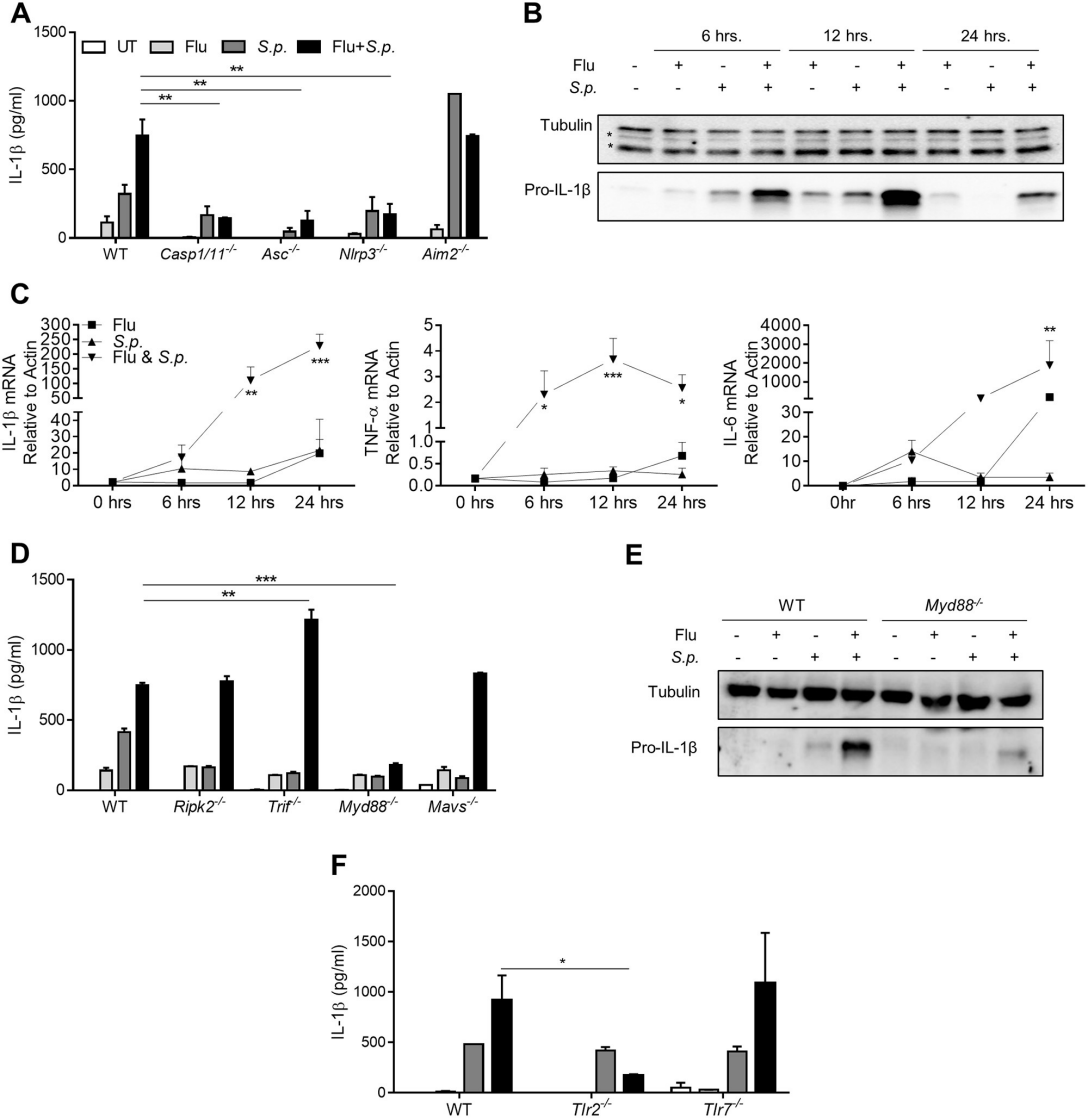
Production of IL-1β during coinfection is NLRP3 and TLR2 dependent. (**A, D, F)** BMDMs from the indicated genotype of mice were infected with a single pathogen or coinfected. Samples of culture supernatant were collected 24 h post-infection and the levels of IL-1β secreted were analyzed by ELISA. **(B, E)** Protein levels of pro-IL-1β were measured using Western blot analysis from samples collected at 6 h, 12 h, or 24 h after the indicated infection. Tubulin was used as a loading control (* indicates non-tubulin bands). **(C)** mRNA from BMDMs samples collected at 6 h, 12 h, or 24 h post-infection with the indicated pathogens were examined for IL-1β, IL-6, and TNF-α gene expression by qRT-PCR. mRNA was normalized relative to β-Actin. Data are pooled from 2–5 independent experiments using n = 2–3 wells per experiment. One-way ANOVA using Tukey’s post hoc analysis was used for statistical comparison (Mean +/- SEM). p values: <0.05 (*), <0.01 (**), <0.001 (***).

We next examined inflammasome activation by generating BMDMs from WT mice or mice deficient in inflammasome genes *Asc*^*-/-*^, *Casp1/11*^*-/-*^, *Nlrp3*^*-/-*^ or *Aim2*^*-/-*^. Following coinfection, *Asc*^*-/-*^, *Casp1/11*^*-/-*^, and *Nlrp3*^*-/-*^ BMDMs had significantly decreased IL-1β levels compared to WT cells (Fig 2A). However, BMDMs lacking AIM2 were not significantly different from WT cells during coinfection (Fig 2A), and they had IL-1β levels that were enhanced during *S*.*p*. infection alone (Fig 2A).

In addition, we examined pro-IL-1β expression to determine if increased signaling through PRRs during coinfection enhances priming signals. WT BMDMs were again infected with PR8 and *S*.*p*. alone or coinfected 3 hours apart. In samples collected 6 h, 12 h or 24 h after initial infection, we observed pro-IL-1β expression was enhanced during coinfection compared to singly infected samples (Fig 2B and S1 Fig). Pro-IL-1β expression was due to enhanced IL-1β mRNA (Fig 2C). In fact, mRNA levels of several cytokines were enhanced, including TNF-α, and IL-6 mRNA, in coinfected cells compared to single infected cells (Fig 2C). Overall, coinfection enhances transcriptional activation of cytokine genes.

We next examined the signaling pathways upstream that would regulate gene expression. During coinfection, the NOD2-RIPK2 pathway would respond to *S*.*p*. peptidoglycan fragment muramyl di-peptide (MDP), the RIG-I-MAVS pathway would respond to IAV uncapped RNA, and TLRs 2, 3, 7 and 9 would respond to their various ligands and activate TRIF or MYD88. Because all of these PRR pathways can facilitate NF-κB activation and cytokine gene expression through their adaptor proteins, we determined which pathways are involved in IL-1β production during coinfection by infecting BMDMs derived from WT, *Ripk2*^*-/-*^, *Trif*^*-/-*^, *Myd88*^*-/-*^ or *Mavs*^*-/-*^ mice. Intriguingly, *Trif*^*-/-*^ BMDMs had higher IL-1β levels than WT BMDMs, suggesting TRIF signaling may play a regulatory role during coinfection (Fig 2D). Importantly, only coinfected *Myd88*^*-/-*^ BMDMs had significantly reduced IL-1β compared to coinfected WT BMDMs (Fig 2D and 2E and S2 Fig). Finally, we found that *Tlr2*^*-/-*^ BMDMs had significantly impaired IL-1β production during coinfection compared to WT cells (Fig 2F), demonstrating that a TLR-2-MYD88 signaling pathways primes pro-IL-1β during coinfection.

### Pathways regulating IL-1β and the inflammasome *in vivo* during coinfection

**Fig 3:**
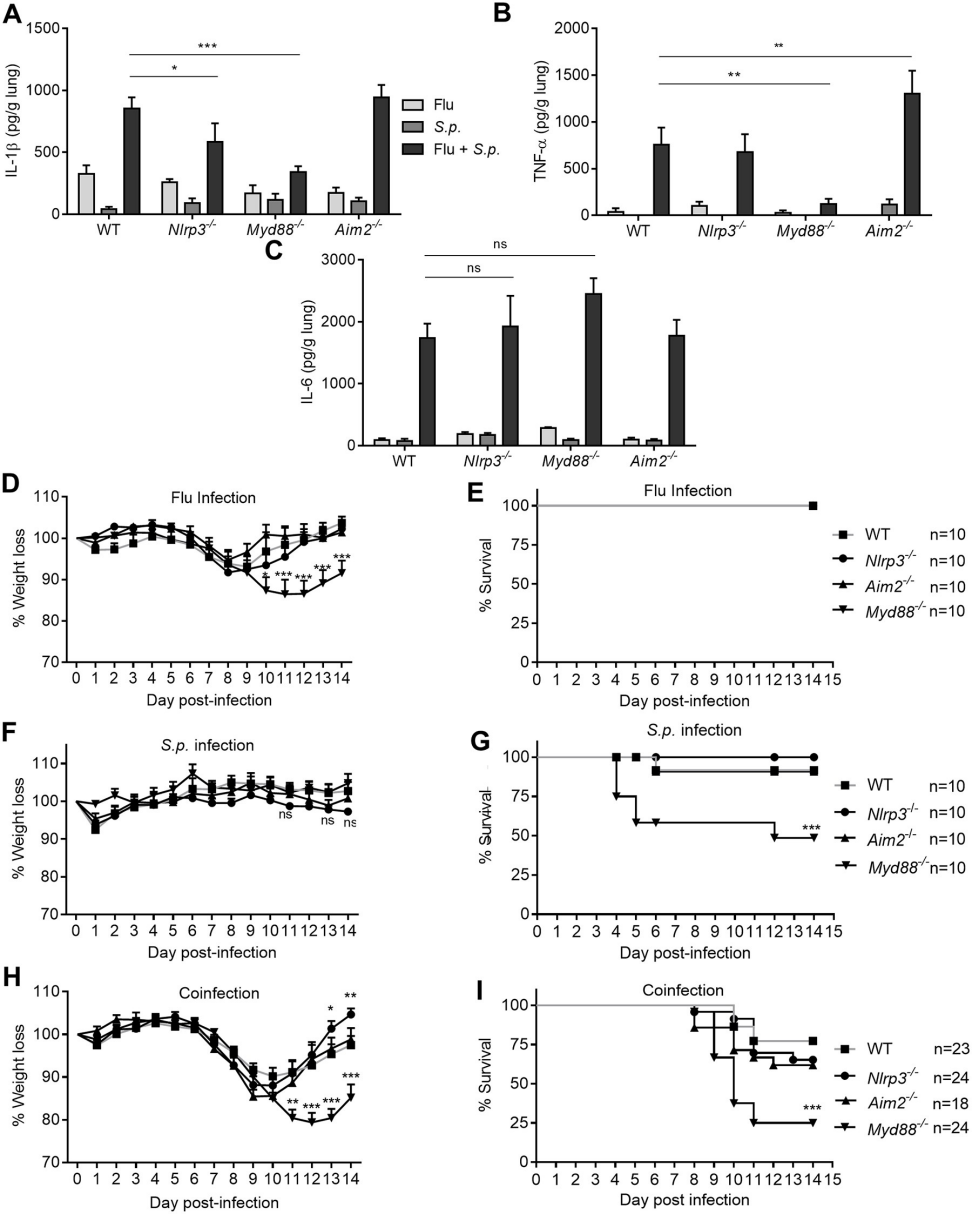
Production of IL-1β *in vivo* is dependent on MYD88 and NLRP3. **(A-C)** Indicated cytokine levels were examined in whole lung homogenates on day 9 post-PR8, day 2 post-*S*.*p*. or day 2 post-coinfection. **(D-I)**. Weight loss and mortality in mice infected with PR8 alone, *S*.*p*. alone, or PR8-S.p. coinfection. **(A-C)** Data are representative of two experiments, n = 5–7 mice per group per experiment. One-way ANOVA using Tukey’s post hoc analysis was used for statistical comparison (Mean +/- SEM). **(D-I)** Data are combined from 2–3 experiments, total n is indicated. Two-way ANOVA using Tukey’s post hoc analysis was used for statistical comparison for weight loss (Mean +/- SEM) and Kaplan-Meier Survival Plot and LogRank Test for survival data. ns: not significant, p values: <0.05 (*), <0.01 (**), <0.001 (***).

Mice were infected with a non-lethal dose of 125 PFU of PR8 on day 0 and then mock infected or coinfected with a non-lethal dose of 1000 CFU *S*.*p*. on day 7. Another group of mice were singly infected with *S*.*p*. on day 7. Similar to infection in BMDMs, lungs from coinfected WT mice showed increased production of IL-1β, TNF-α, and IL-6 compared to PR8 or *S*.*p*. single infection of WT mice (Fig 3A–3C). Compared to WT coinfected mice, deficiency in either *Nlrp3*^*-/-*^ *or Myd88*^*-/-*^ had significantly decreased levels of IL-1β, and *Myd88*^*-/-*^ mice also had significantly lower TNF-α levels (Fig 3A–3C). Although *Myd88*^*-/-*^ mice lost more weight during single infection with PR8, there was no difference in mortality (Fig 3D and 3E), yet in the case of *S*.*p*. single infection, significant mortality was seen (Fig 3F–3G). During coinfection, *Myd88*^*-/-*^ mice had higher weight loss and mortality than WT mice (Fig 3H–3I). *Aim2*^*-/-*^ mice displayed a similar weight loss and mortality to WT mice (Fig 3H–3I). Finally, although *Nlrp3*^*-/-*^ mice had similar mortality compared WT mice, their weight recovered earlier than any other genotype of mice (Fig 3H–3I).

### Less bacteria in peripheral organs in *Nlrp3*^*-/-*^ mice is associated with improved weight recovery

**Fig 4:**
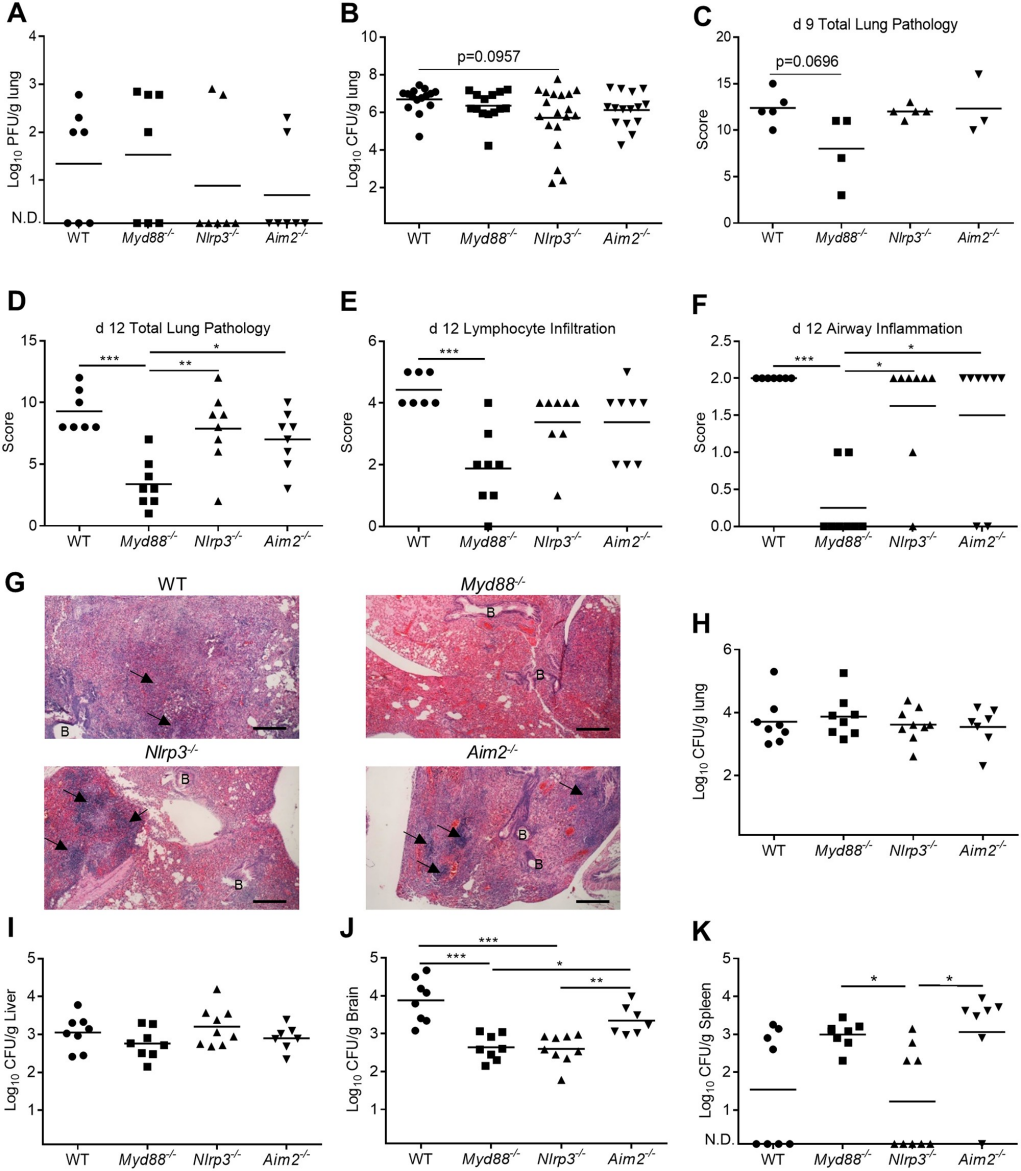
Pathogen titers and lung pathology in coinfected mice. **(A-B)** Viral and bacterial titers in whole lung homogenates of coinfected mice on day 9 (day 2 after coinfection). **(C)** Total Lung pathology scores from coinfected mice on day 9 (day 2 after coinfection). **(D-F)** Lung pathology scores from coinfected mice on day 12 (day 5 after coinfection). **(G)** Lung histology images on day 12 (day 5 after coinfection). Original magnification is 400x (B, indicates Bronchi; Arrow, indicates region of lymphocyte infiltration). **(H-K)** Bacterial titers in whole lung, Liver, brain and spleen homogenates of coinfected mice on day 12 (day 5 after coinfection). Data are representative of one-two independent experiments, n = 5–9 mice per group per experiment. One-way ANOVA using Tukey’s post hoc analysis was used for statistical comparison (Mean +/- SEM). ns: not significant, p values: <0.05 (*), <0.01 (**), <0.001 (***).

To understand the improved weight recovery seen in *Nlrp3*^*-/-*^ mice and the increased susceptibility of *Myd88*^*-/-*^, we examined viral and bacterial titers during coinfection. By day 9 (2 days post-coinfection), PR8 was cleared from the lungs of most mice, and there were no significant differences in viral titers (Fig 4A). *S*.*p*. titers were high on day 9 (2 days post-coinfection). However, there was a trend for *Nlrp3*^*-/-*^ mice toward lower bacterial burden in the lungs, but this did not reach statistical significance compared to WT mice (p = 0.0957) (Fig 4B). Examination of lung pathology on day 9 showed that *Myd88*^*-/-*^ mice had slightly reduced overall lung pathology that approached significance (p = 0.0696) compared to WT mice (Fig 4C). This was mainly due to reduced cellular infiltrates into the lung (data not shown). Because differences in weight between WT, *Myd88*^*-/-*^ and *Nlrp3*^*-/-*^ mice did not occur until day 10 or later, we examined lung pathology and pathogen loads in mice on day 12 (5 d post-coinfection). There was improved overall lung pathology in *Myd88*^*-/-*^ mice on day 12, particularly with respect to airway inflammation and lymphocyte numbers (Fig 4D–4G), but this was independent of lung bacterial numbers, as all genotypes of mice had similar lung bacterial loads on day 12 (Fig 4H). Bacterial numbers in the liver were also similar between all genotypes of mice (Fig 4I). Intriguingly, *S*.*p*. numbers in the brain were lower in both *Nlrp3*^*-/-*^ and *Myd88*^*-/-*^ mice compared to WT and *Aim2*^*-/-*^ mice (Fig 4J). However, *Myd88*^*-/-*^ mice and *Aim2*^*-/-*^ mice had more bacteria in the spleen than WT or *Nlrp3*^*-/-*^ mice (Fig 4K). Thus, earlier weight recovery in *Nlrp3*^*-/-*^ mice was associated with lower bacterial burden in both brain and spleen compared to other genotypes of mice, but was independent of lung pathology.

## Discussion

The invasion of bacteria like *S*.*p*. in IAV infected hosts is linked to increased death rates during pandemic outbreaks, such as the 1918 “Spanish Flu”, where pneumococcus was found in samples collected from infected individuals [53–56]. Coinfections also occur during seasonal influenza epidemics to varying degrees [34, 57]. Previous reports show that pro-inflammatory cytokines, such as TNF-α, IL-6, and type I IFN, increase during coinfection [21, 22]. IL-6 and type I IFN display detrimental effects but TNF-α is protective during coinfection [21, 22]. Thus, an improved understanding of the role for various cytokines and immune cells during coinfection is needed to understand this disease.

Some studies have examined IL1 receptor signaling during bacterial coinfection with IAV. Bansal et al. recently reported that *Il1r1*^*-/-*^ mice are more susceptible to IAV-*S*.*p*. coinfection due to decreased alveolar macrophage numbers in *Il1r1*^*-/-*^ mice [45]. However, Bansal et al. also found that *Casp1*^*-/-*^ mice had similar survival to WT mice during IAV-*S*.*p*. coinfection [45]. This is in agreement with our findings where in *Nlrp3*^*-/-*^ mice had similar survival to WT mice (Fig 3I). However, these results suggest the inflammasome and IL-1β are not responsible for alveolar macrophage survival or mouse survival, or that IL-1β and IL-1α play redundant roles. Alternatively, Bansal et al. hypothesized an inflammasome independent mechanism for producing IL-1β [45]. A second group, Robinson et al., reported that *Il1r1*^*-/-*^ mice are more susceptible to coinfection with IAV and a different bacteria, *S*.*a*., due to impaired Th17 responses [46]. However, there is a notable difference between our findings and those reported for *Il1r1*^*-/-*^ mice infected with IAV-*S*.*a*. IAV-*S*.*a*. coinfection reduces IL-1β levels temporarily for the first 24 hours [46]. In our experiments, coinfection with IAV and *S*.*p*. only results in enhanced IL-1β levels. In a subsequent study, Robinson et al. treated IAV-*S*.*a*. infected mice with the NLRP3 inflammasome inhibitor MCC950 and found decreased *S*.*a*. numbers in the lungs, but similar survival compared to WT mice [58]. Similar to this second report by Robinson et al. [58], we found that IAV-*S*.*p*. coinfected *Nlrp3*^*-/-*^ mice have lower bacterial numbers, but this was mainly in the brain and spleen, not the lung (Fig 4J and 4K).

Although these studies have contributed to our understanding of IL-1 signaling during coinfection, how the inflammasome is activated during IAV-*S*.*p*. coinfection was not well understood. Our findings demonstrate increased expression of pro-IL-1β during coinfection with IAV and *S*.*p*. is dependent on the TLR2-MYD88 pathway. Furthermore, we demonstrate that *Nlrp3*^*-/-*^ mice or macrophages release less IL-1β than WT controls or *Aim2*^*-/-*^ macrophages and mice. Because *Nlrp3* deletion did not completely eliminate IL-1β production *in vivo*, other inflammasomes or pathways must be involved in IL-1β production *in vivo* during IAV-*S*.*p*. coinfection. One hypothesis is that a combination of NLRP3 and AIM2 contributes to inflammasome activation *in vivo*. Alternatively, other proteases in the lung, such as neutrophil elastase, may activate IL-1β [58]. Although *Nlrp3*^*-/-*^ mice had only partially decreased IL-1β levels, we did observe improved weight recovery in these mice compared to WT mice. However, instead of reducing overt inflammation, as we originally hypothesized, *Nlrp3*^*-/-*^ mice had similar inflammation to WT mice, in the lungs. Instead, on day 12, *Nlrp3*^*-/-*^ mice had low bacterial burden in both brain and spleen, which was not observed in any other genotype of mice. This suggests the inflammasome and IL-1β may inhibit specific responses required for bacterial control, as eliminating NLRP3 improves bacterial burden. However, the mechanisms involved will require further investigation. As mentioned above, a previous report showed that mice treated with the NLRP3 inflammasome inhibitor MCC950 had decreased *S*.*a*. numbers during IAV coinfection, and this would support this hypothesis [58]. Interestingly, *Myd88*^*-/-*^ mice in our experiments displayed decreased levels of IL-1β in the lung, decreased bacteria in the brain, and decreased lung pathology, yet they were more susceptible to coinfection. This would suggest that overt inflammation and tissue damage are not the only factors involved during coinfection [59].

## Supporting information

S1 Fig Original western blots for Fig 2B. All western blots in their uncropped format used to make conclusions presented in Fig 2B for this paper are included here.
(TIF)

S2 Fig Original western blots for Fig 2E. All western blots in their uncropped format used to make conclusions presented in Fig 2E for this paper are included here.
(TIF)

S1 File Supporting data for graphs. This file contains all the original data points used to generate the graphs throughout the manuscript.
(XLSX)
