# Supplementary figures and images for: Enhanced IL-1β production is mediated by a TLR2-MYD88-NLRP3 signaling axis during coinfection with influenza A virus and Streptococcus pneumoniae
Source: PLoS One. 2019 Feb 22;14(2):e0212236. doi: 10.1371/journal.pone.0212236 (PMC6386446; doi:10.1371/journal.pone.0212236)

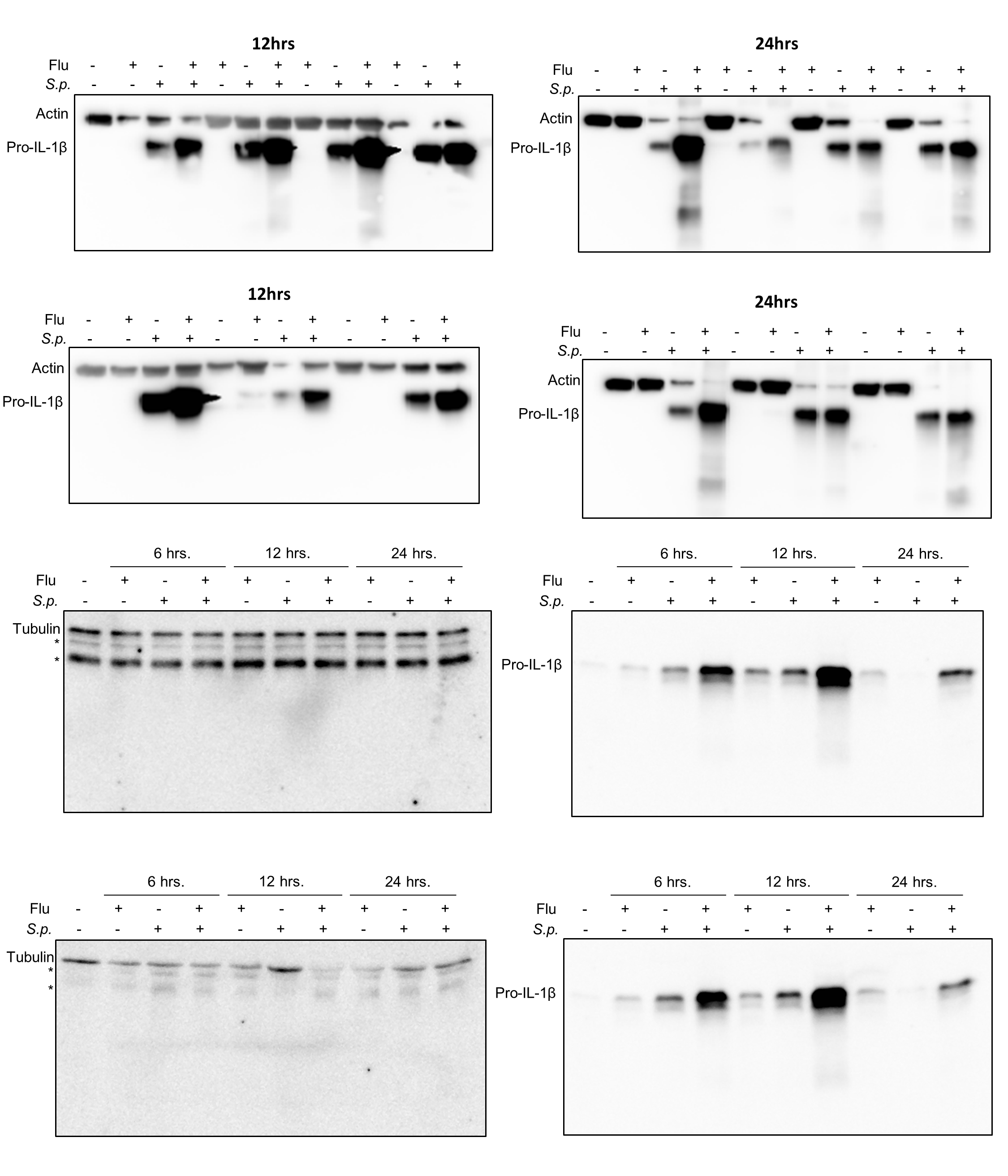

Supplement: S1 Fig — All western blots in their uncropped format used to make conclusions presented in Fig 2B for this paper are included here. (TIF) [file pone.0212236.s001.TIF]

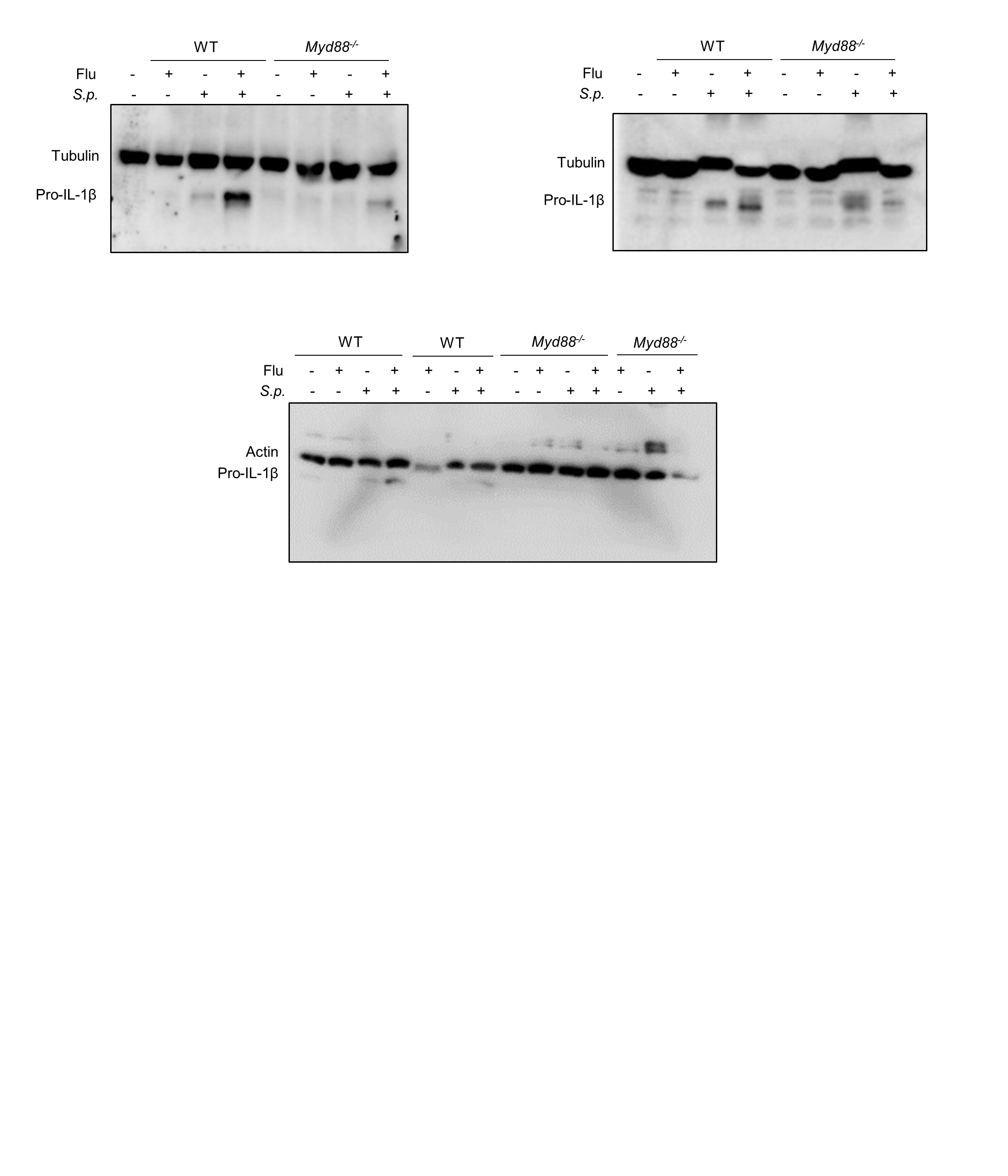

Supplement: S2 Fig — All western blots in their uncropped format used to make conclusions presented in Fig 2E for this paper are included here. (TIF) [file pone.0212236.s002.TIF]
